# Structure of the SigE regulatory network in *Mycobacterium tuberculosis*

**DOI:** 10.3389/fmicb.2024.1407500

**Published:** 2024-05-30

**Authors:** Laura Cioetto-Mazzabò, Davide Sorze, Fedora Babic, Francesca Boldrin, Greta Segafreddo, Roberta Provvedi, Riccardo Manganelli

**Affiliations:** ^1^Department of Molecular Medicine, University of Padova, Padova, Italy; ^2^Department of Biology, University of Padova, Padova, Italy

**Keywords:** *Mycobacterium tuberculosis*, sigma factor, stress response, host-parasite interaction, pathogenesis, gene regulation

## Abstract

SigE is one of the main regulators of mycobacterial stress response and is characterized by a complex regulatory network based on two pathways, which have been partially characterized in conditions of surface stress. The first pathway is based on the induction of *sigE* transcription by the two-component system MprAB, while the second is based on the degradation of SigE anti-sigma factor RseA by ClpC1P2, a protease whose structural genes are induced by ClgR. We characterized the dynamics of the SigE network activation in conditions of surface stress and low pH in *Mycobacterium tuberculosis*. Using a series of mutants in which the main regulatory nodes of the network have been inactivated, we could explore their hierarchy, and we determined that MprAB had a key role in the network activation in both stress conditions through the induction of *sigE*. However, while in conditions of surface stress the absence of MprAB totally abrogated *sigE* induction, under low pH conditions it only resulted in a small delay of the induction of *sigE*. In this case, *sigE* induction was due to SigH, which acted as a MprAB backup system. The ClgR pathway, leading to the degradation of the SigE anti-sigma factor RseA, was shown to be essential for the activation of the SigE network only following surface stress, where it showed an equal hierarchy with the MprAB pathway.

## 1 Introduction

**Figure 1:**
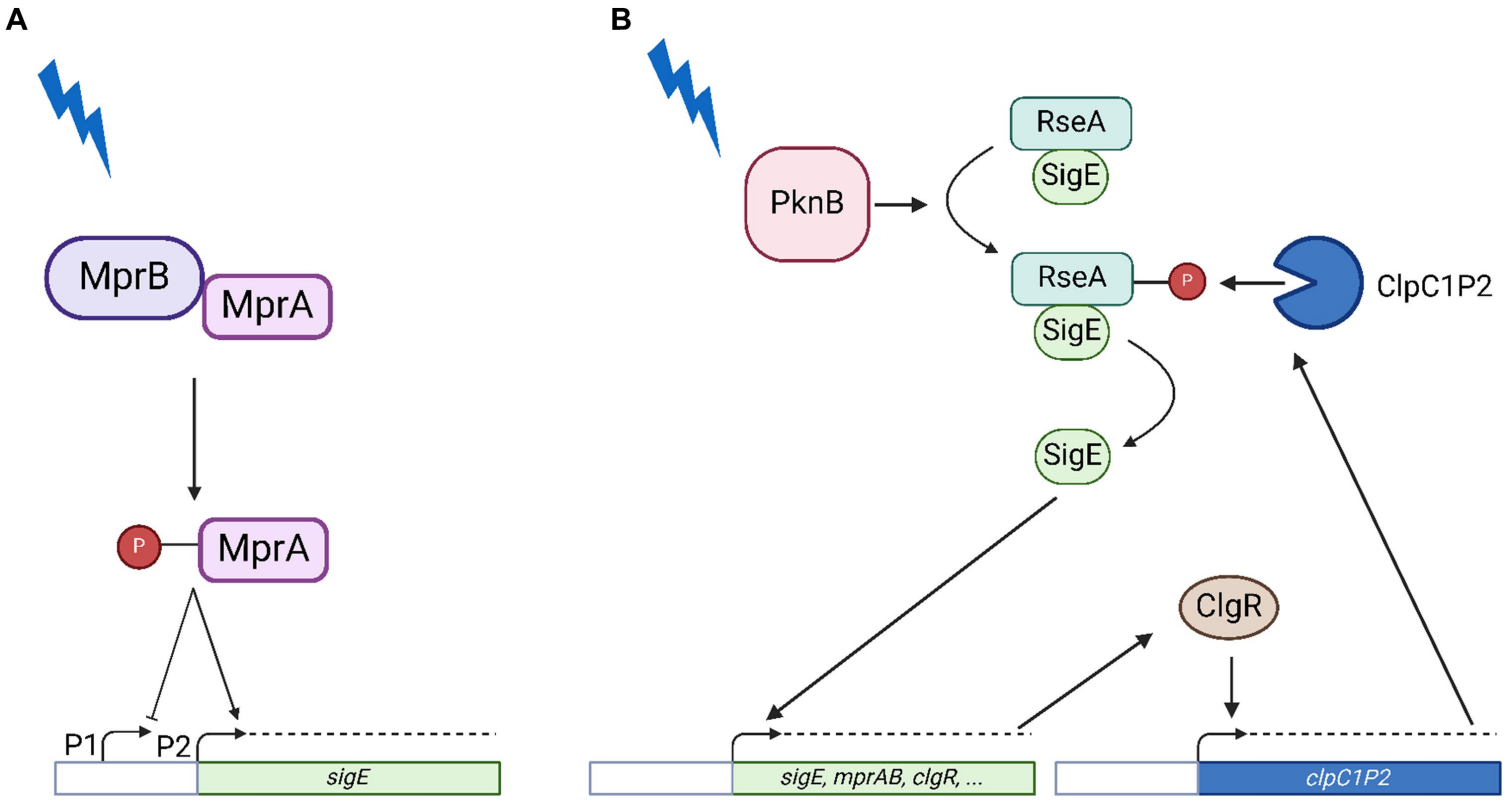
Model of SigE network activation in conditions of surface stress. **(A)** Transcriptional activation of the *sigE* promoter by the two-component system MprAB. Following surface stress MprB phosphorylates MprA which recognizes its operators in the *sigE* upstream region activating transcription from P2 while inhibiting transcription from P1. **(B)** Post-translational activation of SigE. Following surface stress PknB phosphorylates RseA, which becomes sensitive to proteolytic degradation by ClpC1P2, whose structural genes are under transcriptional control of the pleiotropic regulator ClgR, a member of the SigE regulon. Further explanations can be found in the text. Created with BioRender.com.

The extracytoplasmic function (ECF) sigma factor SigE is considered one of the main regulators encoded by the *Mycobacterium tuberculosis* chromosome, required for virulence in macrophages, dendritic cells, mice and guinea pigs (Manganelli et al., 2004, 2023; Casonato et al., 2014; Troudt et al., 2017). Transcription of its structural gene *sigE* is induced upon exposure to hostile conditions such as surface stress, oxidative stress, low pH, phosphate starvation, drug treatment and intracellular growild typeh (Manganelli et al., 2001; Schnappinger et al., 2003; He et al., 2006; Sureka et al., 2007; Kundu and Basu, 2021). Being involved in the adaptation to so many different conditions, SigE is at the centre of a complex regulatory network and its activity is closely monitored at various levels (Manganelli et al., 2023). First, the expression of *sigE* is driven by three different promoters: the farthest from the transcription start site (P1) is recognized by the main sigma factor SigA, the second (P2) is recognized by ECF sigma factor: SigH (Donà et al., 2008). After exposure to surface stress, MprAB is activated: upon phosphorylation by MprB, the response regulator MprA binds to its operator upstream of *sigE*, turning off P1 in favor of P2 (Donà et al., 2008) (Figure 1A). Other genes belonging to the SigE regulon include its own structural gene and the genes encoding MprAB, thus resulting in multiple positive feedback loops (Manganelli and Provvedi, 2010). Moreover, SigE is regulated at the post-translational level by an anti-sigma factor (RseA), whose coding gene lays immediately downstream to *sigE* and is transcribed independently (Donà et al., 2008; Boldrin et al., 2019). Finally, SigE induces the expression of *clgR*, which encodes a pleiotropic regulator that activates transcription of the genes encoding the ClpC1P2 protease (Estorninho et al., 2010), responsible for the degradation of SigE anti-sigma factor RseA upon its stress-dependent phosphorylation by PknB (Barik et al., 2010) (Figure 1B). This complex regulatory network has been suggested to represent a bistable switch and proposed to be involved in development of dormancy and persistence (Tiwari et al., 2010; Boldrin et al., 2020; Zorzan et al., 2021). Indeed, we showed that a *sigE* null mutant is not only more sensitive to several drugs but is also less prone to develop persisters surviving at high dosages of bactericidal drugs (Pisu et al., 2017). Recently, the activation of the SigE network has also been linked to sensitivity to pyrazinamide (Thiede et al., 2022). Despite the detailed characterization of this network, the hierarchy of its different regulatory nodes is still unknown; as well as the physiology of the system in conditions of low pH. In this paper, we sought to fill existing gaps by analyzing the activity of the *sigE* network in a series of *M. tuberculosis* mutants where either *mprAB* or *clgR* have been deleted, or in which RseA carries a mutation preventing its phosphorylation and thus its degradation by ClpC1P2 (Barik et al., 2010). To analyze the functionality of the partners involved, we monitored the expression level of various genes at different time points after exposure to surface stress and to low pH. We chose these two conditions since they are both relevant during the infectious process since they mimic the conditions encountered by the bacterium during the infection of the macrophages. The chosen genes were *sigB*, whose expression almost completely depends on SigE, and therefore represents a perfect reporter to study its activity (Manganelli et al., 2001); *sigE* and *clgR*, both subjected to SigE transcriptional regulation, at least under some physiological conditions; *clpP2*, encoding one of the subunits of the ClpC1P2 protease, whose transcription is controlled by ClgR (Barik et al., 2010) and *rseA*, encoding SigE-specific anti-sigma factor.

## 2 Materials and methods

### 2.1 Bacterial strains, media, and growth conditions

*M. tuberculosis* H37Rv was grown in either Middlebrook 7H9 liquid medium or Middlebrook 7H10 solid medium (Difco) supplemented with 0.2% glycerol (Sigma Aldrich), 0.05% Tween 80 (Sigma Aldrich) and ADN (2% glucose, 5% bovine serum albumin, 0.85% NaCl). *M. tuberculosis* liquid cultures were grown in roller bottles at 37°C. Plates were incubated at 37°C in sealed plastic bags. When required, antibiotics were added at the following concentrations: kanamycin 20 μg/mL and hygromycin 50 μg/mL.

To perform the experiments under surface stress condition, cultures were grown until the early log phase (OD_540/600_ = 0.4), and the samples were collected at different time points (5, 15, 30, 60, and 90 min) after the addition of sodium dodecyl sulfate (SDS) 0.05% to extract RNA.

To perform the experiments under acidic pH conditions, cultures were grown in Sauton’s medium (3.67 mM K_2_HPO_4_, 4 mM MgSO_4_, 30 mM L-asparagine, 0.18 mM ferric ammonium citrate, 5 mM citric acid, 4 mM glycerol, 0.1 mL 1% zinc sulfate, 0.05% Tween 80) until OD_540/600_ = 0.8. The cultures were then split in two to allow the resuspension at pH 6.8 and 4.5 and the samples were collected at different time points (15, 30, 60, 90 min). The pH of the minimal medium was adjusted with 10 M NaOH and antibiotics were added when required.

Bacterial viability in the cultures exposed to stress and used to collect RNA did not show any statistically significant variation during the entire course of the experiment (van Wijk et al., 2023) (Supplementary Figure S1). The bacterial strains used in this work are listed in Table 1.

**Table 1:** *M. tuberculosis* strains used in this work.

Name	Relevant genotype	Reference
H37Rv	Parental strain	Lab collection
TB522	*H37Rv* Δ*clgR*	This work
TB552	*H37Rv* Δ*mprAB*	This work
TB340	*H37Rv* Δ*sigE_*Δ*rseA*	Boldrin et al. (2019)
TB509	*H37Rv* Δ*sigE_*Δ*rseA::sigE::rseA_T39A_*	This work
TB572	*H37Rv* Δ*clgR::clgR*	This work
TB573	*H37Rv* Δ*mprAB::mprAB*	This work

### 2.2 DNA manipulations and electroporation

Recombinant DNA techniques to construct the plasmid required to create mutant and complemented *M. tuberculosis* strains were performed according to standard procedures, and *Escherichia coli* DH5α was used as initial host. When required, antibiotics were added at the following concentrations: kanamycin (Sigma-Aldrich) 50 μg/mL, hygromycin (Invitrogen) 150 μg/mL. DNA restriction and modifying enzymes were purchased from New England BioLabs and used according to the instructions provided by the manufacturer. Preparation of electrocompetent cells and electroporation were performed as previously described (Maciąg et al., 2007). All primers and plasmids used for the cloning procedures are listed in Supplementary Tables S1, S2.

### 2.3 Construction of null mutants in *Mycobacterium tuberculosis*

*M. tuberculosis* null mutant TB522 (Δ*clgR*) was constructed using the two-step homologous recombination technique based on the pNIL/pGOAL system (Parish and Stoker, 2000). Briefly, two DNA regions, one upstream and one downstream *clgR* were amplified by PCR and sequentially cloned into pCR-Blunt II-TOPO (Invitrogen) as DraI/StuI and StuI/NotI fragments, respectively. A region upstream *clgR* (991 bp) was amplified by the primer pair RP893/RP894, while a region downstream *clgR* (999 bp) was amplified by the primer pair RP895/RP896. The DraI/NotI fragment was then subcloned into p1NIL previously cut with the restriction enzymes ScaI and NotI. The resulting vector was named pLCM16. A *lacZ-sacB-hyg* cassette from pGOAL19 was then introduced as a PacI fragment into pLCM16 to obtain the final suicide plasmids pLCM17 (Supplementary Figure S2).

After electroporation with the final suicide plasmid, cells were plated onto 7H10 plates containing kanamycin, hygromycin and X-gal to select the first crossover event, whose occurrence was later confirmed by PCR. Blue colonies (deriving from mutants in which the plasmid had integrated onto the chromosome) were streaked on no drug plates of 7H10 to allow the occurrence of the second recombination event. Streaks were then collected and plated on 7H10 plates containing X-gal and sucrose. White colonies were collected and analyzed for Km sensitivity (Supplementary Figure S3). Finally, Km-sensitive colonies were tested by PCR with primers flanking the deleted region to demonstrate the presence of the deletions (Supplementary Figure S4).

A *mprAB* null mutant in *M. tuberculosis* was constructed using ORBIT (Oligonucleotide-mediated Recombineering followed by Bxb1 Integrase Targeting) (Murphy et al., 2018). The target-specific oligonucleotide RP2151 was designed to contain the flanking regions of the *mprAB* operon (specifically, the first 60 bp were selected across the *mprA* start codon and the last across the *mprB* stop codon) interrupted by the mycobacteriophage L5 *attP* site sequence as indicated in the ORBIT protocol (Murphy et al., 2018) (Supplementary Figure S5). *M. tuberculosis* H37Rv was transformed with the plasmid pKM461, to obtain the acceptor strain TB545. TB545 was grown up to OD_540_ = 0.8, treated with anhydrotetracycline (ATc) for 8 h, and subsequently with 2 M glycine for 16 h. The culture was then electroporated with 1 μg of RP2151 and 200 ng of payload plasmid pKM464. After a 24 h recovery period at 37°C in 2 mL 7H9 ADN, cells were plated onto 7H10 ADN Hyg plates and incubated at 37°C for at least 3 weeks. The resulting colonies were plated in 7H10 medium enriched by sucrose allowing the loss of pKM461. Genetic analysis was performed and a hygromycin resistant colony was selected and checked by PCR (Supplementary Figure S4). The final strain was named TB552.

### 2.4 Construction of a strain of *Mycobacterium tuberculosis* with a T39A mutation in RseA

Site-directed mutagenesis with the QuikChange^®^ II XL Site-Directed Mutagenesis Kit (Agilent) was exploited to introduce a T39A mutation into *M. tuberculosis* RseA according to the manufacturer’s instructions. A StuI fragment including *rseA*, *sigE*, and 535 bp upstream *sigE* was produced using the primer couple RP1917/RP1918 and cloned into pCR-Blunt II-TOPO (Invitrogen), generating the template plasmid pFRA240. Mutagenic primers RP1919 and RP1920, each complementary to opposite strands of the vector, were both designed to contain a mismatch that mutates the ACC codon encoding the T39 of RseA into the alanine codon GCC. The mutated plasmid was amplified by Pfu Ultra HF DNA polymerase using the mutagenic primers, digested with DpnI, and subsequently transformed into DH5α cells to obtain pFRA243. The StuI fragment was then extracted from pFRA243 and inserted into pMV306 resulting in pFRA244. Finally, pFRA244 was electroporated in the previously described *sigE-rseA* null mutant of *M. tuberculosis* TB340 (Boldrin et al., 2019) to obtain the strain TB509.

### 2.5 Construction of *Mycobacterium tuberculosis* complemented strains

DNA fragments including *clgR* or *mprAB* and 500 bp upstream their transcriptional start sites were cloned separately in the integrative plasmid pMV306 following manufacturers instruction of the NEBuilder^®^ HiFi DNA Assembly Cloning Kit (NEB^®^). Both plasmids were initially restricted with EcoRV enzyme. The forward primers were designed to anneal at their 5′ on the EcoRV-end of the plasmids, and at 3′ on a region 500 bp upstream of either *clgR* or *mprAB* transcriptional start site. Accordingly, the reverse primers were designed to anneal at their 5′ half on the end of *clgR* or *mprAB* and at their 3’on the other EcoRV end on the plasmids. A PCR reaction was conducted to amplify *clgR* or *mprAB* with the described primers, and a second enzymatic reaction allowed to fuse the obtained amplicon with the two linearized vectors. The final plasmids were named pLCM22 and pLCM21. The mutant strains thus generated were named TB572 and TB573.

### 2.6 RNA extraction and retro-transcription

Starting from 30 mL of bacterial cultures, 2 mL were centrifuged at 13.000 rpm for 5′ at room temperature for each time point. The pellets were suspended in 1 mL of TRIzol reagent and transferred to 2 mL tubes containing 0.8 mL of 0.1 mm-diameter zirconia/silica beads (BioSpec Products). Cells were disrupted with three 45 s pulses in a Mini-Bead-Beater (BioSpec Products). Chloroform and isoamyl alcohol (24:1 ratio) were added and after 10 min of incubation at room temperature, samples were centrifuged at 13,000 rpm for 30 min at 4°C. The aqueous phase was added to 500 μL of isopropanol and 2 μL of glycogen to allow the precipitation of nucleic acids. Samples were incubated overnight at −20°C. This step was repeated twice. The RNA pellets were washed twice with 300 μL of 75% ethanol and air dried. RNA pellets were resuspended in 2 μL of DEPC-treated water and quantified with Nanodrop (Thermo Fisher). RNA samples were then retro-transcribed to first strand cDNA with M-MLV Reverse Transcriptase (Invitrogen) following manufacturer instructions and conserved at −20°C.

### 2.7 Real-time qPCR

Quantitative reverse transcription real-time qPCR (RT-qPCR) was performed on a 7000 Sequence Detection System (Applied Biosystems) using PowerUP SYBR Green Master Mix (Applied Biosystems). Quantitative analyses were performed as previously described using *sigA* cDNA as internal invariant control (Manganelli et al., 1999). The presence of significant DNA contamination was excluded including RNA samples that had not been reverse transcribed in all the experiments and not accepting samples in which the DNA contamination was higher than 1,000 fold the amount of gene specific cDNA. For each sample, melting curves were collected and analysed to confirm the purity of the amplification products (i.e., the absence of aspecific amplification products). Experiments were performed at least three times, starting from independent biological samples. Sequences of the primers for RT-qPCR are listed in Supplementary Table S3.

## 3 Results and discussion

### 3.1 Mutant strains construction

To study the transcription dynamics and hierarchy of the regulators involved in the SigE regulatory network in *M. tuberculosis*, we constructed mutant strains in which either the gene encoding the pleiotropic regulator ClgR or those encoding the two-component system MprAB were deleted (Table 1). Moreover, we constructed a *M. tuberculosis* mutant in which the *rseA* gene was mutagenized to replace the threonine residue phosphorylated in response to surface stress by PknB (T39) with an alanine residue (RseA_MtbT39A_) (Table 1). In wild type conditions, the phosphorylation of this residue makes RseA accessible to the ClpC1P2 protease, allowing its degradation with subsequent release of SigE upon surface stress (Barik et al., 2010). Consequently, in the mutant strain RseA is no longer able to release SigE in response to surface stress (see Materials and methods for details).

### 3.2 Regulation of the SigE network in conditions of surface stress

In a first experiment, *M. tuberculosis* wild type strain H37Rv was exposed to SDS-mediated surface stress. At different time points after stress exposure, samples were collected for RNA extraction, and then analyzed by RT-qPCR using *sigA* cDNA as internal invariant control (Manganelli et al., 1999) to evaluate the differential expression of selected genes in relation to stress response.

**Figure 2:**
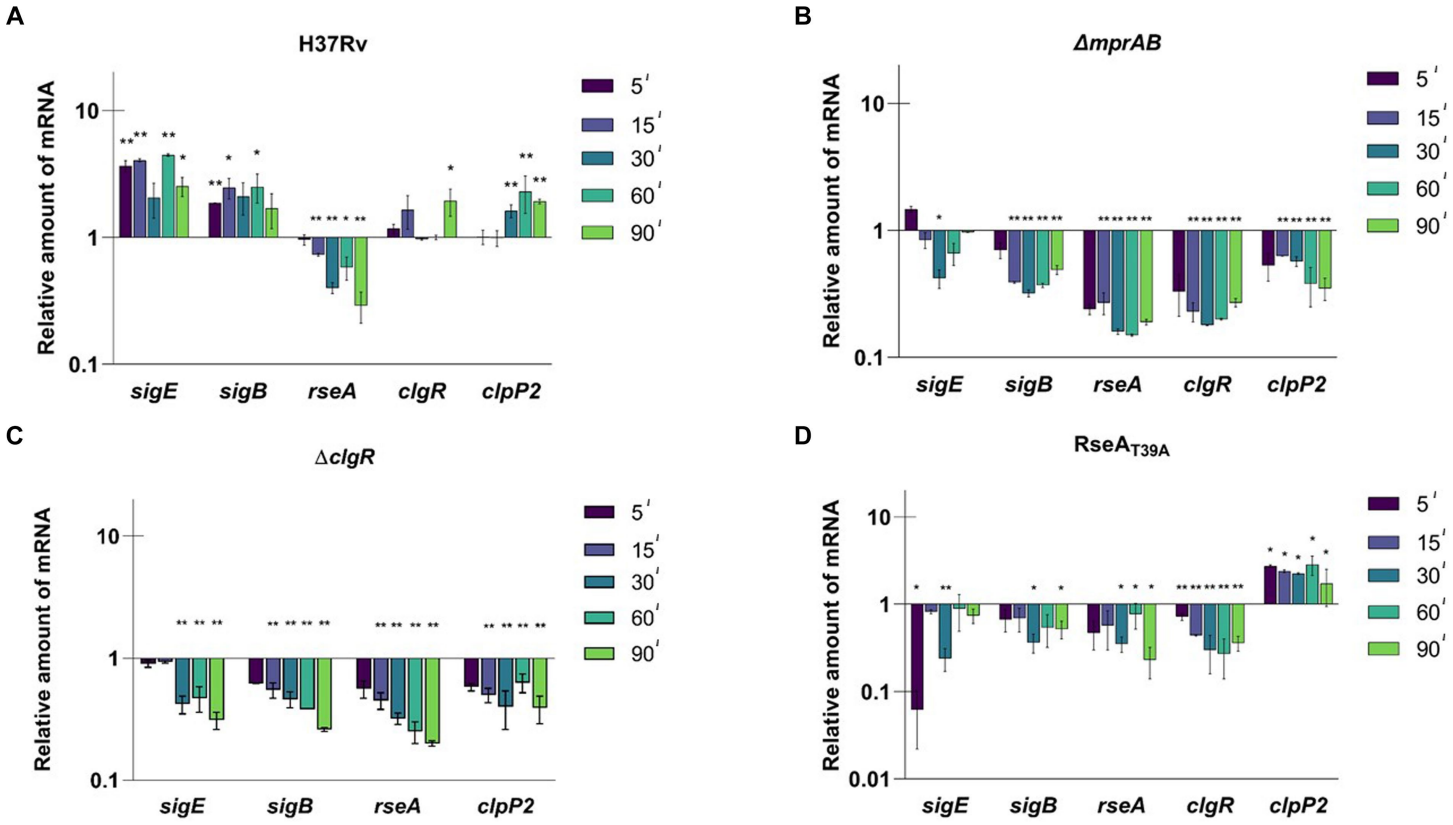
Relative amounts of mRNA levels of specific genes after surface stress in different *M. tuberculosis* strains. Values are expressed as the ratio between the number of cDNA copies detected by RT-qPCR in samples obtained from exponentially growing cultures of the different mutants collected at 5, 15, 30, 60, 90 min after exposure to SDS 0.05% compared to the untreated control. **(A)** H37Rv; **(B)** TB552 (Δ*mprAB*); **(C)** TB522 (Δ*clgR*); **(D)** TB509 (RseA_T39A_). Data were normalized to the level of *sigA* cDNA that represented the internal invariant control. The reported values derive from at least three independent experiments. ^*^p < 0.05 and ^**^p < 0.005 versus untreated control (student’s *t*-test).

As clearly visible in Figure 2A, *sigE* was fully induced after 5 min of exposure to SDS and remained induced until the end of the experiment (90 min). Accordingly, given its dependence on SigE (Manganelli et al., 2001), also *sigB* was upregulated for the whole course of the experiment. *clgR* and *clpP2* mRNAs remained instead at their baseline expression levels in the first part of the experiment, to show statistically significant induction only at later time points. Finally, *rseA* expression was clearly repressed.

The experiment was then repeated with the different mutants. In the strain lacking the two-component system MprAB, SigE network was not activated in response to surface stress, with the amount of the mRNA of the selected genes decreasing as soon as 5 min post exposure, suggesting a critical role of this two-component system in activating the SigE network under these conditions (Figure 2B). Comparable results were observed for the *clgR* mutant strain (Figure 2C), demonstrating that both MprAB and ClgR are essential to activate the SigE network in response to surface stress. The reintroduction of wild type *clgR* and *mprAB* in the chromosomes of the mutants restored the induction of *sigB* and *sigE* (Supplementary Figures S6A,B).

Finally, we analyzed the mutants in which the anti-sigma factor RseA contained a mutation preventing its phosphorylation by PknB and thus its degradation by ClpC1P2 (*rseA_T39A_*). In this strain the mRNA level of the selected genes either remained unchanged or decreased except for that of *clpP2*, which showed a significant increase (Figure 2D). An explanation as to why *clpP2* would be induced regardless of a correspondent induction of *clgR*, can be found in the reported evidence that ClgR is regulated at the post-translational level by PspA (Manganelli and Gennaro, 2017). In this scenario, the release of ClgR from PspA would increase the regulator levels in the cytoplasm, with the subsequent effect on *clpP2* transcription. According to this supposition, the same effect should have been visible in the *mprAB* mutant, where no induction of *clpP2* was detected instead. These findings suggest that ClgR requires the presence of MprAB to activate *clpP2* promoter, a hypothesis worth confirming in the future.

### 3.3 Regulation of the SigE network in conditions of low pH

**Figure 3:**
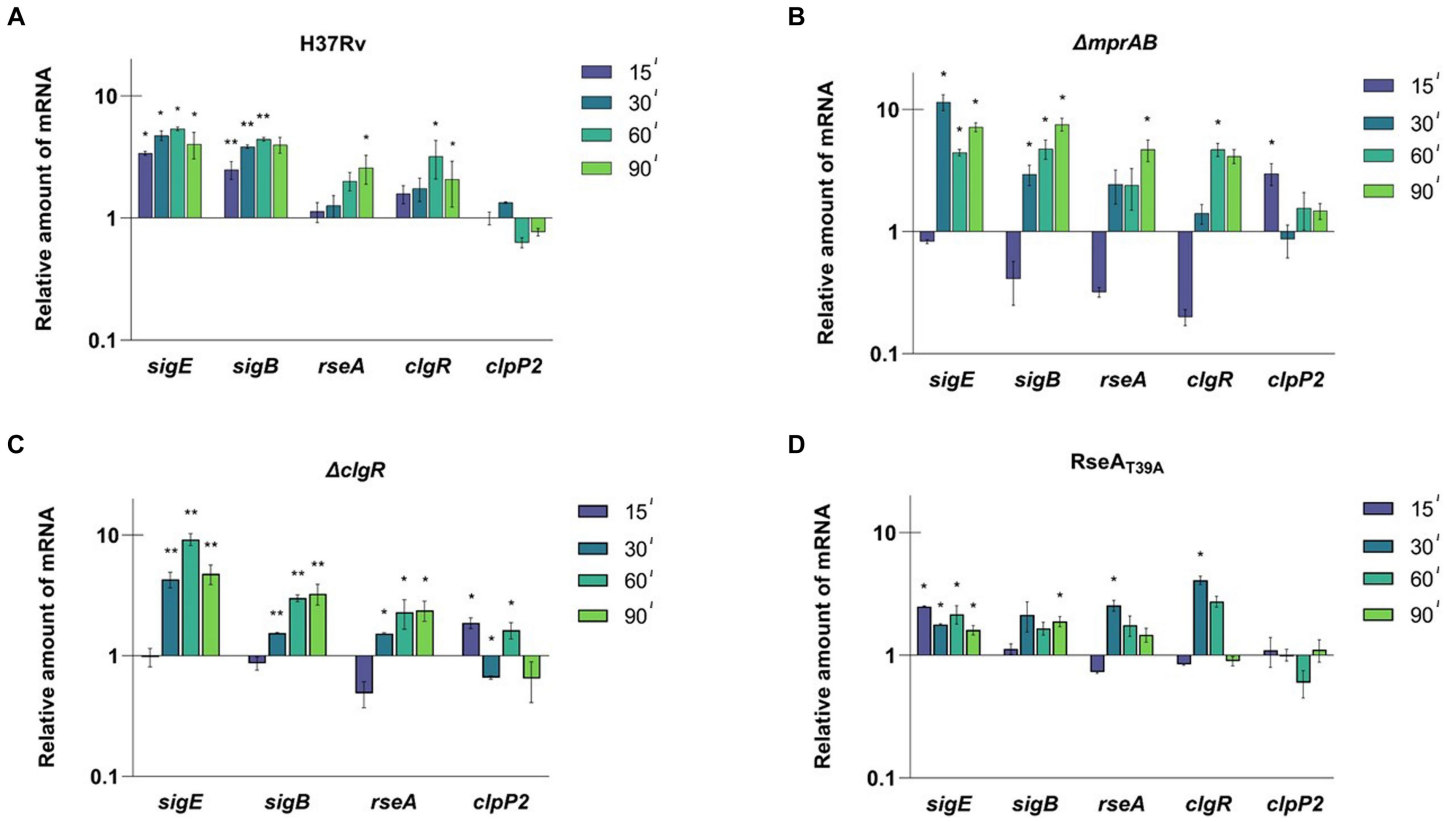
Relative amounts of mRNA levels of specific genes after exposure to low pH in different *M. tuberculosis* strains. The values are expressed as the ratio between the number of cDNA copies detected by RT-qPCR in samples obtained from exponentially growing cultures of the different mutants collected at 15, 30, 60, 90 min after the resuspension in medium at pH 4.5 compared to the samples resuspended in medium at pH 6.8. **(A)** H37Rv; **(B)** TB552 (Δ*mprAB*); **(C)** TB522 (Δ*clgR*); **(D)** TB509 (RseA_T39A_). Data were normalized to the level of *sigA* cDNA that represented the internal invariant control. The reported values derive from at least three independent experiments. ^*^p < 0.05 and ^**^p < 0.005 versus pH 6.8 (student’s *t*-test).

A similar experimental scheme was employed to evaluate the activation dynamics of the SigE network following exposure to low pH. For this purpose, a bacterial culture grown at pH 6.8 was divided into two parts and centrifuged; the two samples were then resuspended, the first in medium at pH 6.8, and the second in medium at pH 4.5. At different time points samples were collected and the level of expression of the selected genes was evaluated by RT-qPCR. In H37Rv we detected a clear time-dependent induction of *sigE*, *sigB*, *rseA* and *clgR*. However, *clpP2* was not induced, confirming that ClgR induction does not necessarily lead to the induction of *clpP2* (e.g., if PspA does not release it) (McGillivray et al., 2014) (Figure 3A). Notably, *rseA* was induced, and not repressed as after surface stress, suggesting a different regulation of this gene under diverse conditions, an aspect surely worthy of detailed studies in the future.

The experiment was then repeated with the described mutants strains. After 15 min of exposure to low pH, *sigE* was not induced in the *mprAB* mutant, while the mRNA levels of *rseA*, *sigB*, and *clgR* strongly decreased when compared to T0. The only gene to show a small, but statistically significant mRNA increase was *clpP2* (Figure 3B). However, after 15 additional minutes of exposure (T30) *sigE*, *sigB*, and *rseA* showed a strong induction, while *clpP2* mRNA returned to its basal level of expression. The delayed response of this mutant with respect to the wild type strain suggests that MprAB is involved, even if not essential, in the activation of the SigE-mediated response at low pH.

Also, in the *clgR* and *rseA_T39A_* mutants, the expression profile indicated a clear delay of the response of 15 min compared to the wild type strain, with the only exception of a slight induction of *sigE* at T15 in the *rseA_T39A_* mutant. The reintroduction of wild type *clgR* and *mprAB* in the chromosomes of the mutants restored induction of *sigB* and *sigE* (Supplementary Figures S6C,D). Taken together these data suggest that both MprAB, and the ClgR-RseA pathway are involved, although not essential for network activation under these conditions (Figures 3C,D).

### 3.4 In the absence of MprAB, SigH functions as a backup for *sigE* induction

**Figure 4:**
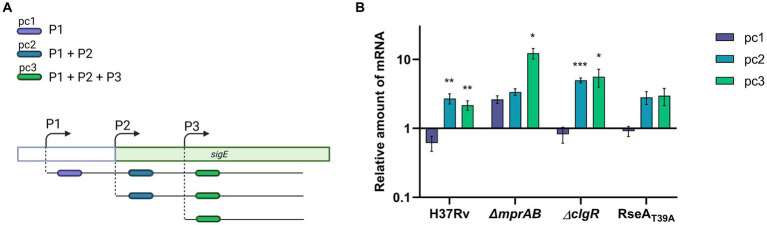
*sigE* promoter usage after exposure to pH 4.5 in different *M. tuberculosis* strains. **(A)** Strategy to study promoter usage: three different primer couples were used to evaluate the variation of transcripts initiated at P1 (pc1), at P1 and P2 (pc2), or from all of the three promoters responsible for *sigE* transcription (P1 + P2 + P3) (pc3). **(B)** Relative amounts of mRNA levels obtained from pc1, pc2 or pc3 in different *M. tuberculosis* strains after exposure to low pH. Values represent the ratio between the number of cDNA copies detected in samples obtained from the cultures exposed 1 h to pH 4.5 and the number of cDNA copies detected in samples obtained from the cultures exposed 1 h to pH 6.8. The values were normalized to the level of *sigA* cDNA, representing the internal invariant control. The reported values derive from at least three independent experiments. ^*^p < 0.05, ^**^p < 0.005, and ^***^p < 0.0005 versus pH 6.8 (student’s *t*-test). Created with BioRender.com.

As already mentioned, in *M. tuberculosis sigE* is transcribed from three different promoters depending on SigA (P1), SigE (P2) and SigH (P3). Furthermore, the MprAB two-component system has an opposite effect on P1 and P2, as binding of phosphorylated MprA to its operators deactivates P1 in favour of P2 (Donà et al., 2008). To better understand the regulation of *sigE* after exposure to low pH, we decided to determine from which of these promoters *sigE* was induced in the different mutants. To this end, we used a method we had previously developed to determine the role of such promoters in *sigE* transcription following surface stress (Donà et al., 2008). Briefly, we performed a RT-qPCR using three primer pairs, the first specific for P1-derived transcripts (pc1), the second able of detecting both P1 and P2-derived transcripts (pc2) and a third capable of detecting transcripts from all three promoters (pc3) (Figure 4A). As clearly visible in Figure 4B, following exposure to low pH the amount of transcripts detected in the wild type strain using pc1 slightly decreased, while those detected by the two other primer couples increased comparably, demonstrating that *sigE* induction under these conditions depends on P2, which requires both SigE and MprAB. These data suggest that, differently from what was hypothesized in the previous paragraph, MprAB is indeed the main actor in *sigE* induction at low pH in the wild type strain, but that in its absence *sigE* is induced using a different mechanism. In accordance with this hypothesis, induction of *sigE* transcription would not start from P2 in the *mprAB* mutant. Indeed, after exposure to low pH, we detected a minor induction from P1 and a strong induction from P3 in this mutant (Figure 4B). The absence of P1-driven induction in the wild type strain was probably due to the repression by phosphorylated MprA. A strong induction of P3 instead strongly suggests the involvement of the redox sensing sigma factor SigH (Manganelli et al., 2002), which has previously been shown to regulate this promoter (Donà et al., 2008) (Figure 4B).

It has been reported that long exposure to reductive stress caused by low pH leads to increased SigH activity due to the production of reactive oxygen species (ROS), which oxidize its anti-sigma partner RshA allowing the release of the sigma factor (Coulson et al., 2017; Baker et al., 2019). We can hypothesize that immediately after exposure to low pH, the SigE network activated by MprAB in the wild type strain intervenes to mitigate stress and adapt to this environment by preventing the formation of ROS. Only if low pH exposure lingers for enough time, ROS are produced in sufficient amounts to activate SigH. In the *mprAB* mutant the absence of activation of the SigE network allows a fast production of ROS and an early SigH activation. A similar explanation has been recently proposed for SigH activation in a *sigE* mutant grown in low phosphate (Baruzzo et al., 2023).

The same experiment was performed in the *clgR* and the *rseA* mutants. In both strains *sigE* induction in response to low pH was similar to that obtained in the wild type strain and was due to transcription from P2 (Figure 4B). The fact that in these strains the activation of the network was only slightly delayed suggests that under these conditions RseA degradation plays a minor role in SigE release. Since it is well known that SigE-RseA interaction, like SigH-RshA interaction, is inhibited under oxidative conditions (Barik et al., 2010), we can hypothesize that under low pH conditions the release of SigE from RseA is mainly due to the presence of pH-induced ROS and not to ClpC1P2-mediated degradation of RseA. Further studies will be necessary to address this issue.

## 4 Conclusion

### 4.1 Surface stress

**Figure 5:**
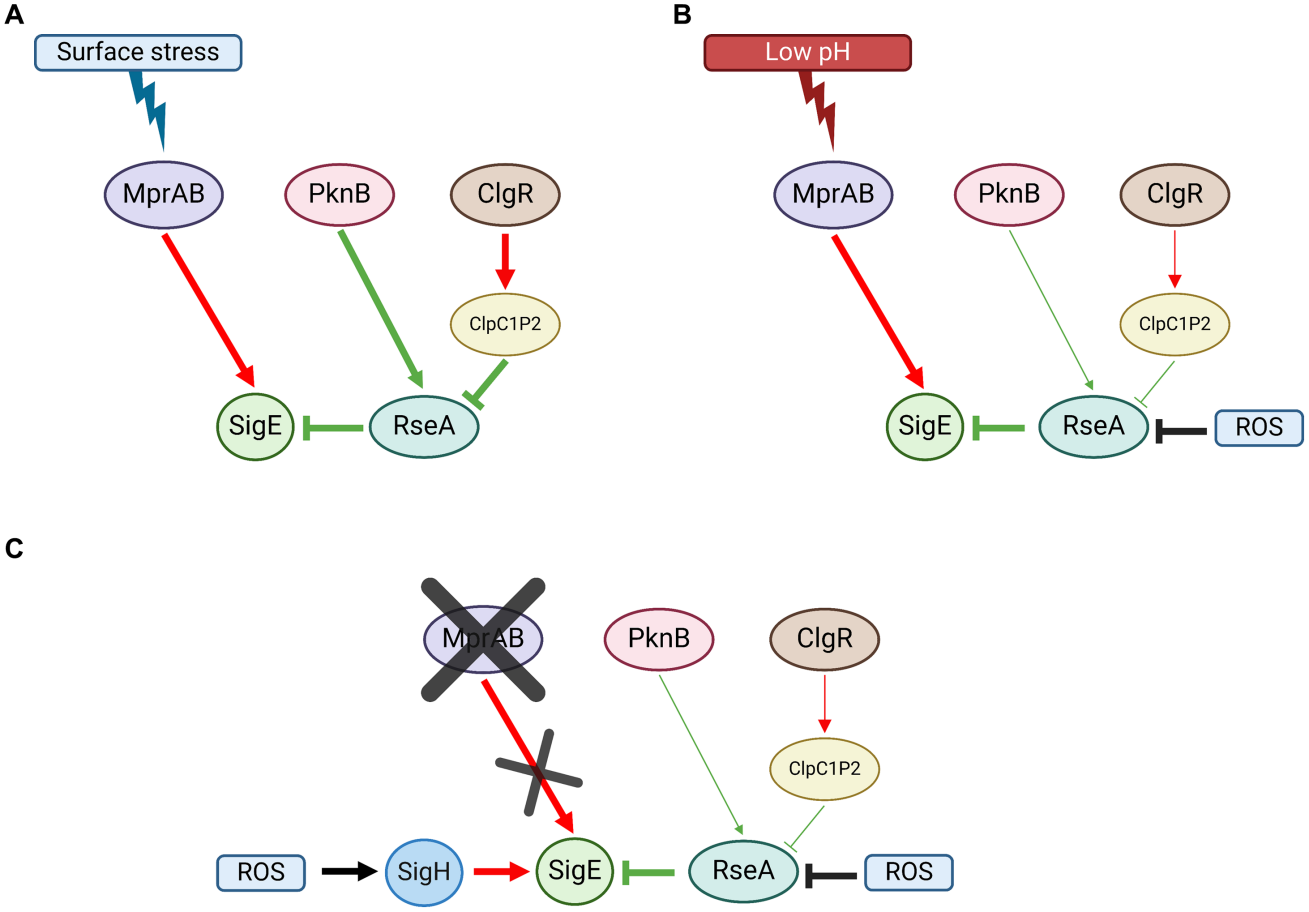
SigE network activation model in *M. tuberculosis*. **(A)** Surface stress; MprAB-dependent induction of *sigE*, RseA phosphorylation by PknB, and its degradation by ClpC1P2 following the induction of its structural genes by ClgR, are all equally essential for the activation of the network. **(B)** Low pH: MprAB-dependent induction of *sigE* is the main activation mechanism of the network. RseA degradation by ClpC1P2 plays a minor role. RseA releases SigE upon its oxidation due to ROS produced upon exposure to low pH. **(C)** In the absence of *mprAB*, the induction of *sigE* under low pH conditions is due to the alternative sigma factor SigH, which responds to oxidative stress (again produced following exposure to low pH). Red: induction of structural gene; green: post-translational interactions; black: environmental stimuli. Created with BioRender.com.

Under surface stress conditions, MprAB, ClgR, and RseA are equally essential to fully activate the SigE network, suggesting that both activation of P2 by phosphorylated MprA and degradation of RseA by ClpC1P2, after induction of the protease structural genes by ClgR, are essential (Figure 5A).

### 4.2 Low pH

In low pH conditions, neither MprAB nor ClgR nor RseA degradation are essential for the activation of the SigE network. However, we found that in the wild type strain of *M. tuberculosis* as well as in the *clgR* and in the *rseA* mutants, *sigE* induction depended on the MprAB-dependent promoter (P2), suggesting that this two-component system can function as a low pH sensor and represents the main *sigE* switch even under low pH conditions (Figure 5B). Of note, MprB is reported to be negatively regulated by the chaperone DnaK, which binds its extracellular domain under physiological conditions. In the presence of improperly folded proteins in the periplasm, DnaK releases MprB to bind them, thus activating the sensor (Bretl et al., 2014). Since we can hypothesize that not only surface stress (e.g., that induced by detergents), but also an acidic environment might lead to the denaturation of proteins in the periplasmic space, both conditions could result in the activation of MprB. In our experiments, in the mutant lacking MprAB, induction of *sigE* was ensured by the activation of an alternative promoter (P3) dependent on SigH, which acted as a backup system, probably in response to the uncontrolled production of ROS in the mutant after exposure to stress (Figure 5C). A redox potential variation in the cytoplasm might be responsible for conformational changes in RseA which determine its release of SigE, allowing the factor activity in such conditions, rather than a ClpC1P2-driven degradation of the anti-sigma factor.

Considering all proposed evidence, we can conclude that in *M. tuberculosis* (i) MprAB-mediated transcriptional activation of P2 is essential to activate the SigE network both in conditions of surface stress and low pH; (ii) in condition of low pH, transcription from the SigH-dependent promoter P3 can complement the absence of a functional MprAB; (iii) the degradation of RseA after phosphorylation by PknB is as essential as the activation of P2 by MprAB to activate the network when bacteria are exposed to surface stress conditions, while it has a minor role under conditions of low pH exposure; (iv) under low pH conditions, the release of SigE from RseA is most likely mediated by the presence of intracellular ROS and not by its degradation by ClpC1P2.

## Data availability statement

The raw data supporting the conclusions of this article will be made available by the authors, without undue reservation.

## Author contributions

LC-M: Data curation, Investigation, Validation, Visualization, Writing – original draft. DS: Investigation, Visualization, Writing – review & editing. FBa: Data curation, Formal analysis, Investigation, Writing – review & editing. FBo: Conceptualization, Investigation, Supervision, Writing – review & editing. GS: Data curation, Investigation, Writing – review & editing, Conceptualization. RP: Conceptualization, Supervision, Writing – review & editing. RM: Conceptualization, Data curation, Funding acquisition, Project administration, Supervision, Validation, Writing – original draft.
