# Supplementary material for: Structure of the SigE regulatory network in Mycobacterium tuberculosis
Source: Front Microbiol. 2024 May 30;15:1407500. doi: 10.3389/fmicb.2024.1407500 (PMC11173970; doi:10.3389/fmicb.2024.1407500)
Supplement: Supplementary file 1 [file Data_Sheet_1.PDF]

frontiers

Supplementary Material

Table S1. Primers used for the construction of the *M. tuberculosis* mutants

PRIMER	SEQUENCE 5'-3'	TARGET
**RP2151**	TGGGTGTGGCTTTCAACAGTAACTGCACAACTAGGTTGCGCGCGTGGACTGAGATTCCAC**GGTTTGTACCGTACACCACTGAGACCGCGGTGGTTGACCAGACAAACC**ACGATCGTCGTCAACGACAAGAATTCGCACGGACACCAGTGTCGTCGCAGCGCCTGAGAC	*rv0981_rv0982 (mprAB)*<br><br>target oligo specific for *mprAB* (ORBIT strategy). *attP* site in bold
**RP1968**	TTTAAAGGTCATGGCCGGGTCGACAGC	*rv2745c* (*clgR*) upstream region amplification (1006 bp), upper primer
**RP1969**	AGGCCTACGCACCAAAGCCGCCATCAA	*rv2745c* (*clgR*) upstream region amplification (1006 bp), lower primer
**RP1970**	AGGCCTACGCACCAAAGCCGCCATCAA	*rv2745c* (*clgR*) downstream region amplification (923 bp), upper primer
**RP1971**	GCGGCCGCGCCGAACCGTCTGCCCAACT	*rv2745c* (*clgR*) downstream region amplification (923 bp), lower primer

**RP1917**	AAGGCCTGATATCGATGGTGGTCAAGAG	*rv1221_rv1222* (*sigE_rseA*) upstream region amplification (2023 bp), upper primer
**RP1918**	AAGGCCTTTGTCCGTGTCCACCTAGTATC	*rv1221_rv1222* (*sigE_rseA*) upstream region amplification (2023 bp), lower primer
**RP1919**	GACAGGTGCTCGGCGGAACGGAACTGC	Oligo for site directed mutagenesis in *rv1222,* upper primer
**RP1920**	GACAGGTGCTCGGCGGCCAACGGAACTGC	Oligo for site directed mutagenesis in *rv1222,* lower primer
**RP2209**	TTAAATCTAGATATCAGCACGAACCGTTCC	Oligo for Nebbuilder in *rv0981*, upper primer
**RP2210**	GCGGAACCACCACATTCAGGGTGGTGTTTCA	Oligo for Nebbuilder in *rv0981,* lower primer
**RP2211**	TGAAACACCACCCTGAATGTGGTGGTTCCGC	Oligo for Nebbuilder in *rv0982*, upper primer
**RP2212**	CTGCAGCTGGATCCATGCTAGGTTGCGCGCGTGGACTG	Oligo for Nebbuilder in *rv0982,* lower primer

**RP2213**	TTAAATCTA<u>GATATC</u>AACCGCCTGCATTACC	Oligo for Nebbuilder in *rv2745c*, upper primer
**RP2214**	GCAGCTGGATCCATGTTAGGCCACCGCCAG	Oligo for Nebbuilder in *rv2745c*, lower primer

Restriction sites consensus sequences are underlined.

Table S2. Plasmid used to create mutant strains

STRAIN[a]	PLASMID	DESCRIPTION
**TB522**	pLCM16	p1NIL carrying P*mpt64* promoter followed by the homologous regions flanking *clgR*
	pLCM17	pFB15 carrying a *lacZ-sacB-hyg* cassette from pGOAL19
**TB509**	pFRA244	pMV306 carrying *sigE* promoter followed by the genes *sigE* and *rseA* $_{T39A}$
**TB573**	pLCM21	pMV306 carrying *mprAB* gene
**TB572**	pLCM22	pMV306 carrying *clgR* gene

[a] Mutant obtained with the shown plasmids

Table S3. Primers used for quantitative RT-PCR

GENE/REGION	SEQUENCE 5’-3’[1]	REFERENCE
sigA	**F-**CCATCCCGAAAAGGAAGACC<br>**R-**AGGTCTGGTTCAGCGTCGAG	(Boldrin et al., 2019)
sigB	**F-**GTCTATCTGAACGGCATCGG<br>**R-**CCGCCTCGCCATCACGCAC	(Boldrin et al., 2019)
sigE	**F-**CGAAGGCTGGCTACACCGCA<br>**R-**GCAGGTCAGGTCCCAGCC	(Boldrin et al., 2019)
rseA	**F-**CAGTTCCGTTCCACCGAGCA<br>**R-**GGTGGACAACGCGGGATCT	(Boldrin et al., 2019)
clgR	**F-**CTTTGGTGCGTGAGGTCGTTG<br>**R-**ATCGATGAGCACCACCGACAA	This manuscript
clpP2	**F-**CCTGGGCTCGGAGGTGAACG<br>**R-**TTGCCCTTGGTACCTGCCGC	This manuscript
**pc1**	**F-**GCGGACCTGTTGGGGATGAG<br>**R-**CGGTACGCGACGGTAATTCC	(Donà et al., 2008)
**pc2**	**F-**TTTGCGTTGCCGACGGTGAC<br>**R-**CGGTACGCGACGGTAATTCC	(Donà et al., 2008)
**pc3**	**F-**ACGACTTGCCAACTTATTGCAG<br>**R-**TCAGACGGCTCCACCCACT	(Donà et al., 2008)

[1]F: forward primer; R: reverse primer

References

Boldrin, F., Cioetto Mazzabò, L., Anoosheh, S., Palù, G., Gaudreau, L., Manganelli, R., et al. (2019). Assessing the role of Rv1222 (RseA) as an anti-sigma factor of the Mycobacterium tuberculosis extracytoplasmic sigma factor SigE. *Sci Rep* 9, 4513. doi: 10.1038/s41598-019-41183-4

Donà, V., Rodrigue, S., Dainese, E., Palù, G., Gaudreau, L., Manganelli, R., et al. (2008). Evidence of complex transcriptional, translational, and posttranslational regulation of the extracytoplasmic function sigma factor σ^{E} in Mycobacterium tuberculosis. *J Bacteriol* 190, 5963–5971. doi: 10.1128/JB.00622-08

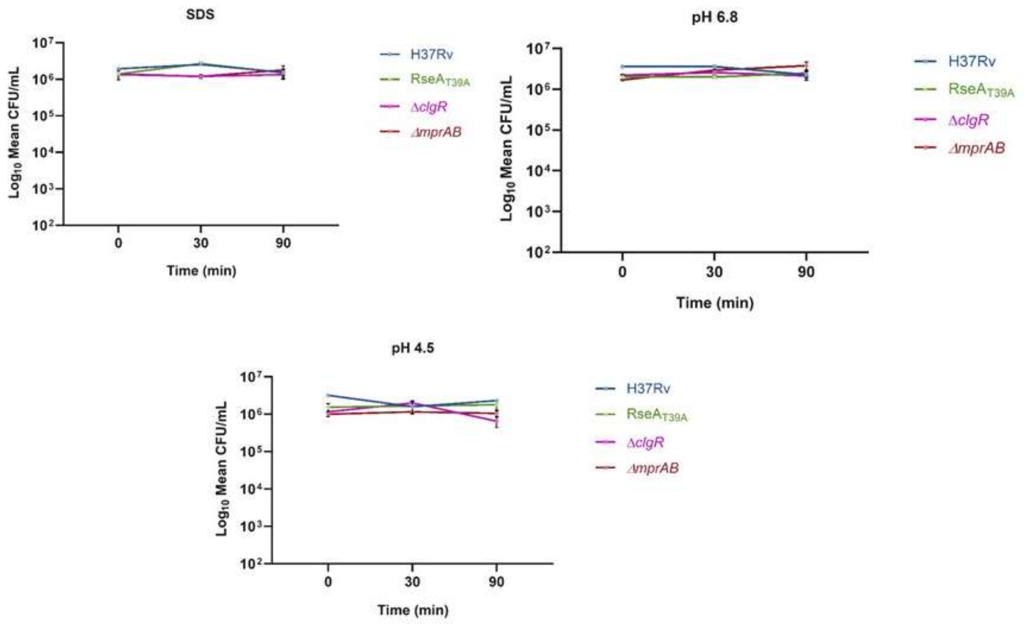

Supplementary Figure 1. Viable counts of H37Rv, and the three mutants used in the study after exposure to the conditions of surface stress and acidic conditions used for RNA extraction. No statistically significant variation was observed among the four strains. The experiment was repeated three times. The graphs shows the results of one representative experiment.

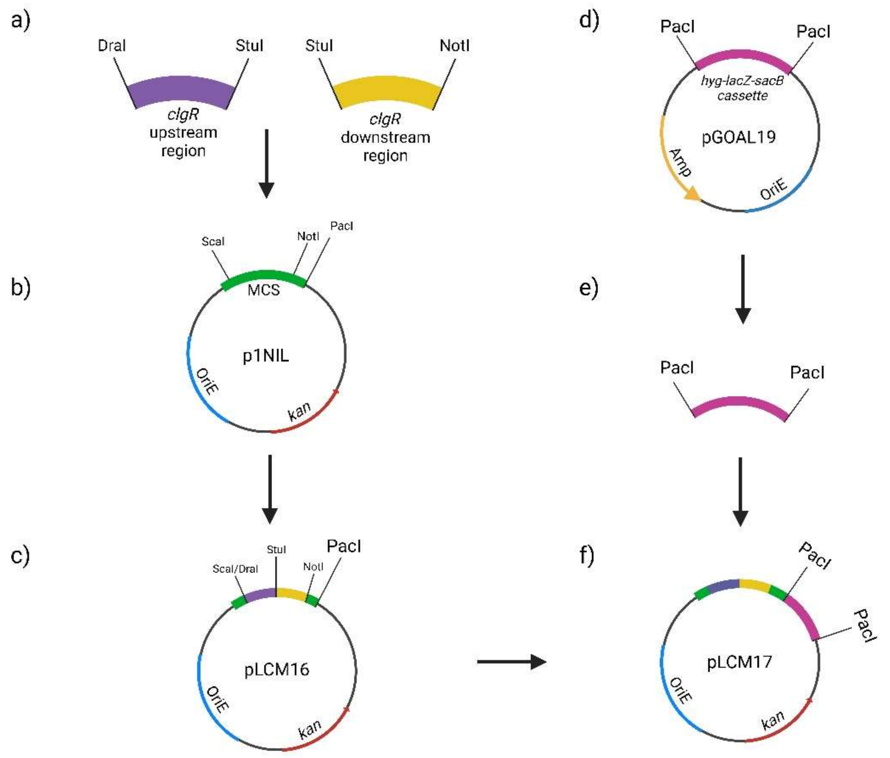

Supplementary Figure 2. Schematic representation of the construction of the vectors used for generating the *clgR* null mutant using the pNIL/pGOAL system (Parish and Stoker, 2000).
a) The upstream and downstream regions of *clgR* were amplified, cloned, and subsequently extracted from pCR-Blunt II-TOPO (Invitrogen) as DraI/StuI and StuI/NotI fragments respectively.
a) Homologous regions were inserted into the p1NIL using ScaI and NotI sites, obtaining the pLCM16 plasmid (c).
d-e) The hyg-lacZ-sacB selection cassette was excised from pGOAL19 and inserted into the pLCM16 plasmid via the PacI site, resulting in the final suicide plasmid pLCM17 (f). Created with BioRender.com.

Reference

Parish, T., and Stoker, N. G. (2000). Use of a flexible cassette method to generate a double unmarked Mycobacterium tuberculosis tlyA plcABC mutant by gene replacement. *Microbiology* 146, 1969–1975. doi: 10.1099/00221287-146-8-1969

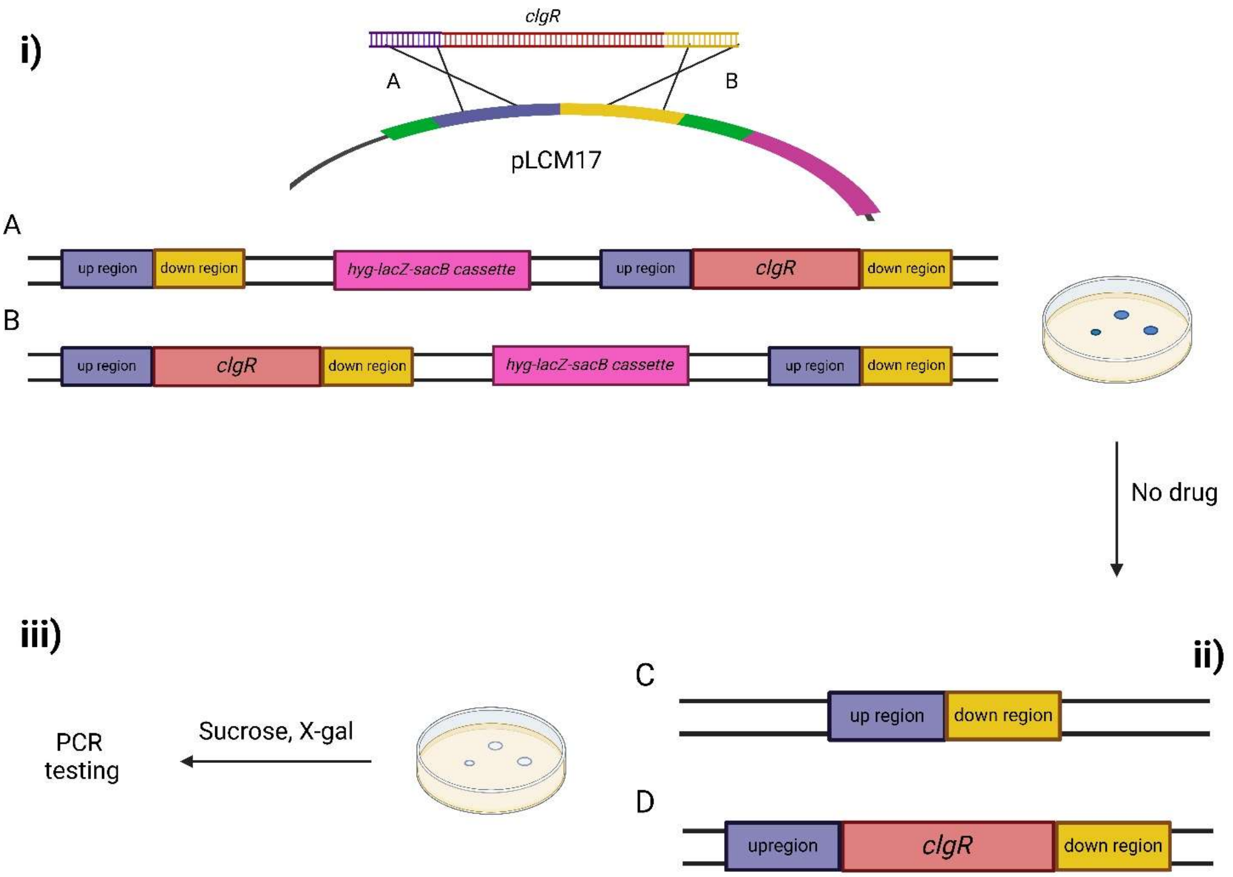

Supplementary Figure 3. Recombination events leading to the construction of the *clgR* mutant. pLCM17 was electroporated into *M. tuberculosis* H37Rv, transformants were selected on plates containing kanamycin, hygromycin, and X-gal. A first recombination event leads to plasmid integration by single crossover, resulting in allele A or B (i). Single blue colonies were grown without selection to allow a second homologous recombination event, leading to the formation of a mixed population: one in which the wild-type allele was reconstituted (D), and the other in which the target gene knockout occured (C) (ii). Selection of the second recombination event was carried over by plating onto plates with sucrose and X-gal. Colonies able to grow in sucrose and unable to metabolize X-gal (white colonies) were collected and PCR-tested to confirm the knockout event (iii). Created with BioRender.com.

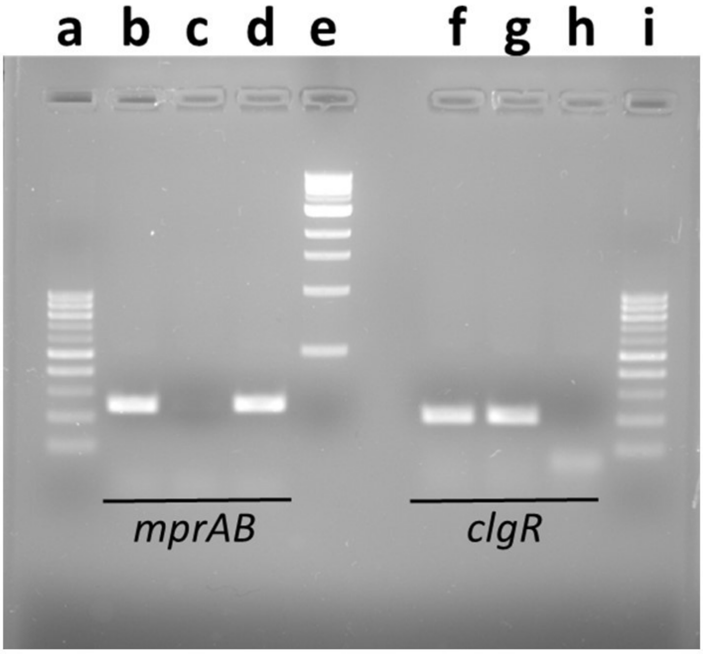

Supplementary Figure 4. Agarose gel to confirm the deletion of *mprAB* and *clgR* in TB552 and TB522, respectively. Lanes **b**, **c**, and **d** were loaded with PCR products from the amplification of the genomes of H37Rv, TB552 and TB522, respectively, all amplified with primers internal to *mprAB*. Lanes **f**, **g**, and **h** were loaded with PCR products from the amplification of the genomes of H37Rv, TB552 and TB522, respectively, all amplified with primers internal to *clgR*. Lanes **a** and **i**: 100bp ladder (Sigma Aldrich); lane **e**: 1Kb ladder (NEB).
Primers internal to *mprAB*:
F-AACGCGCTGGAAGTCTACGT; R-GGCGAACGACATCAGCACAA
Primers internal to *clgR*:
F- CTTTTGTGCGTGAGGTCGTTG; R-ATCGATGAGCACCACCGACAA

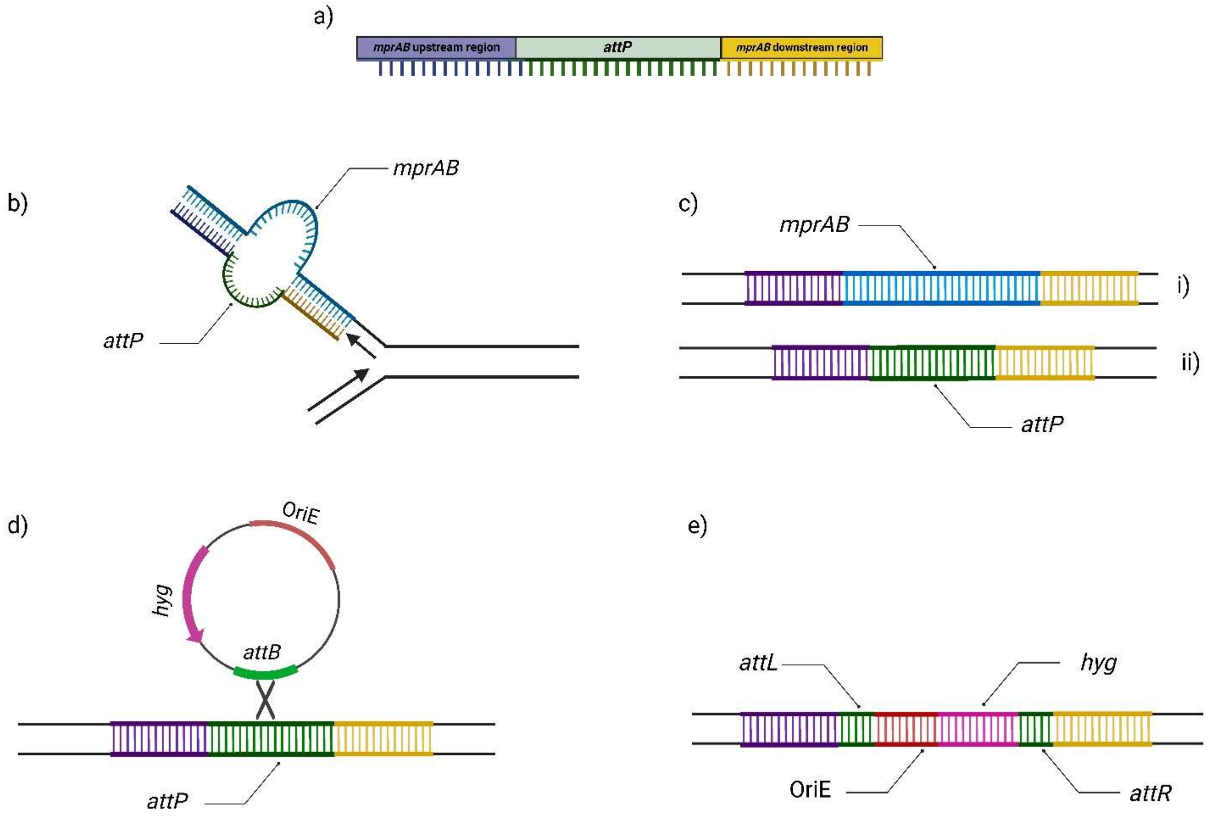

Supplementary Figure 5. Schematic representation of the construction of the null mutant MprAB using ORBIT (Murphy et al., 2018). The specific targeting oligo was designed to contain the flanking regions of *mprAB* upstream and downstream of the *attP* site sequence (a). During DNA replication, the specific targeting oligo pairs via homology regions to the flanking regions of *mprAB* (b). The subsequent replication event leads to the formation of a mixed population of bacteria: one harboring the *wt* allele (i), the other harboring the target gene substitution with the *attP* site (ii) (c).The *attB* site of the plasmid pKM464 recombines with the *attP* site leading to its integration: the presence of the *hyg* selection marker enables the selection of bacteria in which the gene knockout has occurred, resistant to hygromycin (d-e). (modified from Murphy et al, 2018). Created with BioRender.com.

Reference

Murphy, K. C., Nelson, S. J., Nambi, S., Papavinasasundaram, K., Baer, C. E., and Sassetti, C. M. (2018). ORBIT: a new Paradigm for genetic engineering of mycobacterial chromosomes. *mBio* 9, e01467-18. doi: 10.1128/mBio.01467-18

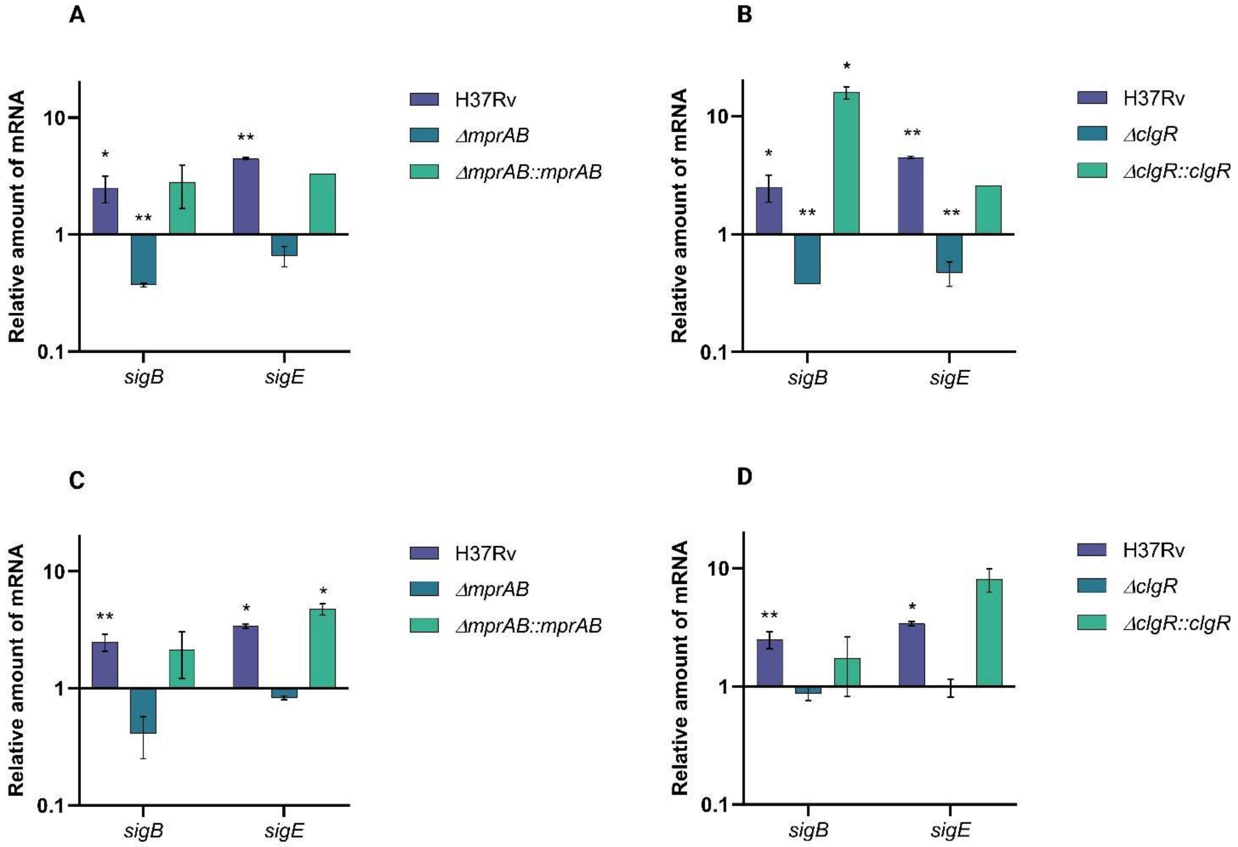

Supplementary Figure 6. Relative amounts of *sigE* and *sigB*-specific mRNA levels in H37Rv, the *mprAB* mutant TB552, the *clgR* mutant TB522 and their complemented strains (TB573 and TB572, respectively). Panels A and B: values are expressed as the ratio between the number of cDNA copies detected by RT-qPCR in samples obtained from exponentially growing cultures of the different mutants collected at 60 minutes after exposure to SDS 0.05% compared to the untreated control, *P < 0.05 **P < 0.005 versus untreated control (Student's *t*-test); panels C and D: values are expressed as the ratio between the number of cDNA copies detected by quantitative RT-PCR in samples obtained from exponentially growing cultures of the different mutants collected at 15 minutes after the resuspension in medium at pH 4.5 compared to the samples resuspended in medium at pH 6.8. The reported values derive from at least three independent experiments, *P < 0.05 **P < 0.005 versus pH 6.8 (Student's *t*-test). The values were normalized to the level of *sigA* cDNA, representing the internal invariant control.
